# Voluntary Vs Nominated Peer Educators: a Randomized Trial within the NoTrap! Anti-Bullying Program

**DOI:** 10.1007/s11121-020-01108-4

**Published:** 2020-03-27

**Authors:** Valentina Zambuto, Benedetta Emanuela Palladino, Annalaura Nocentini, Ersilia Menesini

**Affiliations:** Department of Education, Languages, Interculture, Literature and Psychology, University of Florence, Sede di via di San Salvi 12, Padiglione 26, 50135 Florence, Italy

**Keywords:** Peer-led model, Bullying, Victimization, Peer educator, Effectiveness, Intervention, Defending behaviour

## Abstract

There is a debate in scientific literature about the effectiveness of a peer-led approach to anti-bullying interventions. In order to understand which circumstances and for whom these approaches work best, the present study was carried out within the NoTrap! anti-bullying program. Using a cluster design, classes were randomly assigned into two different peer educator recruitment strategies: volunteering (*N* = 500; 48% females; mean age = 13.5 years, ds = 1.3) vs peer nominated (*N* = 466; 38% females; mean age = 13.9 years, ds = 1.3). Results showed that voluntary peer educators suffered a higher level of victimization, while the nominated ones tended to be more popular and likable. Furthermore, a set of linear mixed-effect models showed that the program was effective in reducing bullying and victimization, and in increasing defending behaviour only in the voluntary recruitment condition. On the contrary, in classrooms under the peer nominated recruitment condition, bullying and victimization remained stable, and defending behaviour increased only for peer educators, but not for their classmates. This implies that the step of peer selection and recruitment must be kept into consideration in developing and validating an intervention, because of its possible impact on the effectiveness of the whole intervention.

**Electronic supplementary material:**

The online version of this article (10.1007/s11121-020-01108-4) contains supplementary material, which is available to authorized users.

## Introduction

### The Effectiveness of Peer-Led Approach in Anti-Bullying Programs

Peer-led approaches to intervention adopt a psycho-educational method where some members of a group are recruited, trained, empowered and reintegrated into their own group to carry out specific activities with their peers (Sun et al. 2018). These approaches could potentially include a wide variety of models and cover different topics. In a recent systematic review (Rose-Clarke et al. 2019), the authors distinguish between “peer education”, where peers aim to increase and influence both adolescents’ knowledge and attitudes; “peer counselling”, where peers provide psychological support; “peer activism”, in which peers carry out campaigns to change health-related policies and “peer outreach”, where peers engage with marginalized adolescents.

These models are widely used in health interventions targeting adolescents and addressing different topics such as substance use, sexual health, HIV prevention and quitting smoking (Abdi and Simbar 2013; Dobson et al. 2017; Layzer et al. 2017; Rose-Clarke et al. 2019; Sun et al. 2018). Peer-led approaches can be very promising for anti-bullying programs as well. The social nature of bullying and cyberbullying in which bystanders play a relevant role (Allison and Bussey 2016; Bastiaensens et al. 2014; Salmivalli et al. 1996) makes this prospective particularly relevant for their prevention.

Nevertheless, in the scientific literature, there has been a debate on the effectiveness of peer-led models (Gaffney et al. 2019b; Lee et al. 2015; Smith et al. 2012; Ttofi and Farrington 2011). The lack of consensus could be explained by referring to the high level of heterogeneity included in the concept of “*working with peers*” (Smith et al. 2012). Different approaches could potentially be associated with varying levels of effectiveness and be more appropriate under certain circumstances or to achieve specific outcomes.

In their meta-analyses on effectiveness of school-based anti-bullying programs, Ttofi and Farrington (2011) affirm that working with peers may have iatrogenic effects and reinforce victimization. For instance, in a study about an ecological anti-bullying program with a peer-led component (Rahey et al. 2002), the authors found that bullying did not decrease after 4 months of implementing the program. Furthermore, in a study about the Friendly Schools project (Cross et al. 2004), both intervention and control groups resulted in increased levels of victimization over time.

On the other hand, there are studies in which peer-led models work. For instance, in the three studies analysed in Lee, Kim and Kim’s meta-analysis (2015) about programs with a peer-counselling component, this strategy achieved a reduction of the perception of bullying (Houlston and Smith 2009), a significant reduction of bullying, an increase in empathic attitudes and self-esteem (Wong et al. 2011), a reduction of aggression and an overall improvement of classroom behaviour (Fonagy et al. 2009). Among these studies, only Houlston and Smith (2009) specified that peer counsellors were volunteers selected by staff and student members of the school council. In all the other studies, the specific way in which peer leaders were selected is unknown (Rahey et al. 2002; Cross et al. 2004; Wong et al. 2011; Fonagy et al. 2009). It is possible that examining this information might help to explain some of the incongruent findings.

### The NoTrap! Program

Among evidence-based interventions framed within the peer education model, there is also the NoTrap! program. It is an online and school-based universal intervention program against bullying and cyberbullying which targets adolescents (Menesini et al. 2017), which has provided evidence of its effectiveness (Palladino et al. 2016). Specifically, in Palladino and colleagues’ studies (Palladino et al. 2016), two independent quasi-experimental trials were carried out in the 2011/2012 and 2012/2013 school years. Results showed that there was a significant decrease in bullying, victimization, cyberbullying and cybervictimization in the experimental group. These changes remained stable even 6 months after the end of the program (Palladino et al. 2016). According to recent meta-analyses, NoTrap! is one of the school-bullying prevention programs that were effective in reducing bullying and victimization among 88 studies (Gaffney et al. 2019b), and one of the most effective in reducing cyberbullying perpetration and cybervictimization among 18 studies (Gaffney et al. 2019a). In general, if we compare the studies considered in the two meta-analyses, NoTrap! is one of the few that have significant effects in two separate trials on all four behavioural outcomes.

### For Whom and Under which Circumstances Peer-Led Models Work

In order to go beyond the debate on the effectiveness of peer-led models in anti-bullying programs, Smith et al. (2012), suggested a deeper focus on the different components involved in a peer-led approach to better understand “*what works*, *for whom and under which circumstances.*” Following this consideration, within the NoTrap! anti-bullying program, the present work will focus on the two latter issues: for whom and under which circumstances the program is effective.

In regard to “*for whom*”, according to Abdi and Simbar (2013), a peer educator is a member of a peer group that receives special training and information, and thus attempts to sustain a positive behaviour change among the group members. This means that a peer-led program should be aimed at empowering not only peer educators, but their peers as well. Nevertheless, in a study by Menesini et al. (2012) on the efficacy of the first version of the NoTrap! program (school year 2009/2010), the authors only found changes in the peer educator group, whereas the program was not effective for their classmates. Consequently, it seems important to evaluate whether the efficacy of the program is generalized to the classmates in the experimental group, and to test whether it is only found in peer educators.

As for “*under which circumstances*”, different contextual factors can potentially affect the effectiveness of a peer-led model, such as the contents and the duration of the training, the trainers’ attitude (more or less directive), the materials and/or activities used to empower peer educators’ skills, the peer educators’ tasks (i.e. support services, mentoring, counselling, mediation/conflict resolution) or the level of autonomy of peer educators in these tasks (Thompson and Smith 2011).

### Peer Educators’ Recruitment Strategy

Among these contextual factors, in the current study, we will focus on an understudied aspect of peer-led models, which is the strategy through which peer educators are recruited. Regarding literature on peer-led anti-bullying programs, most studies do not specify how peer educators have been recruited (Chaux et al. 2016; Fonagy et al. 2009; Twemlow et al. 2005; Rosenbluth et al. 2004; Ortega and Del Rey 2004; Rahey et al. 2002). In some studies, we found peer educators had been chosen by adults (Connolly et al. 2015; Elledge et al. 2010; Houlston and Smith 2009), or via voluntary recruitment (Menesini et al. 2003; Zambuto et al. 2019a). To our knowledge, there are no studies on the impact of peer nomination recruitment in relation to anti-bullying interventions. Understanding how peer educators are chosen on the basis of their classmates’ nominations is very important, given the social nature of bullying and the fact that some peers can play a role in supporting the bully or defending the victim.

The recruitment strategy applied could impact the characteristics of the selected peer educators. For instance, in a study of Houlston and Smith (2009), the school council selected peers for their interpersonal skills, previous experience, approachability, and suitability for the role. Results showed a reduction of bullying perception, but not in behaviour. In a study of Jackson and Campbell (2009), it was found that students selected by teachers to serve as peers for children with autism were more often perceived as popular, likeable and social leaders within the classroom. In a following study based on the same dataset, Campbell and Marino (2009) found that having classes nominate their own peers leads to choosing the most popular, prosocial and class-leading students. Unfortunately, the studies did not imply analyses on the impact of these two recruitment strategies, so we have no information about their effectiveness.

Peer educators chosen by their own peers could be the most popular students (Campbell and Marino 2009). In accordance with Bandura’s theory of social learning, the modelling process could be influenced by the status and prestige of the model. Then, a popular peer educator could have a stronger normative influence over his or her classmates. This means that nominated peer educators, because of their higher social status, could more likely be agents of change within their class, compared to voluntary peer educators.

In relation to voluntary recruitment, in a previous study on NoTrap! peer educators (Zambuto et al. 2019a), the authors examined differences between peer educators, who were all voluntary, and their classmates. Results showed that peer educators were different from their classmates for their higher levels of victimization, defending behaviour, perceived support from friends and prosocial behaviour, with some degree of difference based on their gender. The study was based on the same dataset of a previous one in which NoTrap! was found to be effective in reducing bullying, victimization, cyberbullying and cybervictimization (Palladino et al. 2016). We do not know if the voluntary recruitment has played a role on the effectiveness of the program, because no alternative recruitment strategy to the voluntary one was included in the original study. In any case, these results suggest that self-nomination and volunteer recruitment may allow students who are more sensitive and empathic toward victims to make a change in the group relationship (Zambuto et al. 2019a). We can speculate that voluntary peer educators could be particularly motivated to counteract a problem that they had experienced directly (Zambuto et al. 2019a). In this regard, previous studies found that when peer educators perceived the program as fitting with their personal values, they were more likely to implement it and stay engaged in it over time (Convey et al. 2010; Lorthios-Guilledroit et al. 2018). Besides, a deeper involvement in the problem could make them a more credible source of information for their classmates. On the other hand, it is possible that voluntary recruitment could activate students who have been victimized, and as a result are less empathetic toward bullies. These students may also be seen as “victims” or be unpopular within the class, leading to less uptake of the intervention.

In general, there is a lack of studies on the impact of the peer recruitment strategy on the effectiveness of an intervention. In light of this, in the present study, we will focus and compare two different approaches within the peer-led models: “peer nomination recruitment” and “voluntary recruitment”. It is possible that the effectiveness of a peer-led model depends on the characteristics of the peer educators. Thus, the strategies for the recruitment could become a key factor for the success of the intervention.

## The Present Study

In the present study, we aim to investigate the effect of the peer educators recruitment strategy on the effectiveness of the NoTrap! anti-bullying program, measured as a longitudinal change on the main behavioural outcomes: victimization, bullying and defending behaviour. Starting from this general research goal, we defined an experimental cluster design trial in which we randomly assigned the classes involved in the NoTrap! program to one of the experimental conditions: (A) classrooms in which students voluntarily decide to become peer educators (voluntary recruitment condition—VR); (B) classrooms in which peer educators are nominated by their classmates (nomination recruitment condition—NR).

We can distinguish two different aims:

- Aim 1—investigate whether “voluntary peer educators”, “class-nominated peer educators” and “classmates” (=no peer educators) show different individual and socio-relational characteristics: victimization, bullying, defending behaviour and the sociometrical status (likability and popularity).
- Aim 2—understand “*under which circumstances and for whom*” the NoTrap! program is effective. Specifically, we evaluate whether recruitment and the role played by the students in the program (peer educators vs all the other classmates) affect the effectiveness of the program*.*

## Methods

### Participants and Procedure

One thousand five students (44% females) from 45 classes of 15 secondary schools in Tuscany participated in the NoTrap! program in the 2015/2016 school year. Specifically, there were 432 middle school students (7° and 8° grades) and 573 high school students (9° and 10° grades). The participants’ age ranged from 12 to 18 years old (mean = 13.7 years, ds = 1.34). Seventy-seven per cent of the students belong to the Italian ethnic majority. True to the greater Italian context, the other participants were very diverse. The most consistent ethnic minority groups were 2% Albanian, 1% Romanian and 1% Moroccan, while the remaining 19% of participants came from various countries of the world (each with less of 1% of frequency).

Schools were selected using a self-selection inclusion process, and the classes were selected by the school staff, depending on class teachers’ availability to engage in the program. There were no eligibility criteria. We randomly assigned the classes to two experimental conditions, regardless of the school they belong to: (a) 22 classes in which peer educators were invited to voluntarily assume this role (voluntary recruitment—VR; 518 students) and (b) 23 classes in which peer educators were nominated by their classmates (classmate nomination recruitment—NR; 487 students). This experimental design was adopted in order to control school- and class-level variables by randomly assigning each class to its experimental condition. We also accounted for the nested structure of the data (school and level) in the analyses by using multilevel mixed models.

Both experimental conditions participated in all of the NoTrap! program phases, namely, (1) teacher training, (2) class meeting to raise student awareness, (3) peer educator training and finally (4) activities led by peer educators in class about empathy toward victims and coping strategies to escape from victimization (for a more detailed description of the program, see Menesini et al. 2017 and Zambuto et al. 2019b). The implementation fidelity and dosage were strictly monitored in our experimental design even when the trial was run in real-world conditions. The teachers sent us a report at the end of activities to supply us with evidence on the correct implementation of the final phase led by the peer educators. In both conditions, 100% of classes completed all the program’s phases. All peer educators used the same standardized printed materials during training and for the two class activities. Finally, the same two trainers conducted the awareness meeting and the trainings—with both teachers and peer educators, and they were the teachers’ reference straight from the beginning, as to ensure the highest level of fidelity.

For both experimental conditions at the end of the class meeting to raise student awareness—phase (2), the NoTrap! trainers explained the meaning of being a peer educator in the program. Specifically, it was explained that peer educators had to attend a training session and then carry out activities with their classmates. Then, in the VR condition, the trainers requested for volunteers to assume the role of peer educators. Students who publicly raised their hand were selected. In NR condition, each student wrote the name of a classmate he/she wanted to assume this role on a piece of paper (anonymously); students who received the highest number of nominations became peer educators. Specifically, the number of nominations for each nominated peer educator varied between three and 13. In both conditions, the number of peer educators for each class ranged from three to seven, based on class size. We allowed one peer educator for every 4–5 students in each class.

We had two waves of data collection: November 2015 (T1, wave 1, before starting the NoTrap! program) and May–June 2016 (T2, wave 2, after the end of the two peer-led activities). The questionnaires were administered in class by trained research assistants during school hours.

**Fig. 1:**
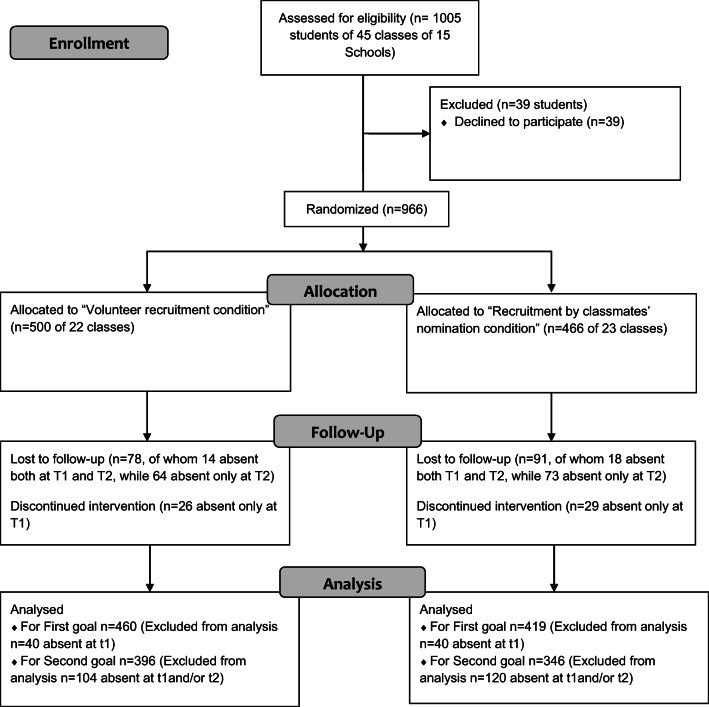
Flowchart of the recruitment and retention of participants in the evaluation

Preliminary informed consent, consisting of initial approval by the School Principal and the class council, was requested. Once permission was gained from schools, informative letters were sent to all students and to their parents, explaining the study, the intervention aims and requesting the parents’ consent for their child’s participation. Ninety-six per cent of the target sample received parents’ approval to participate in the study and in the intervention. Our final sample consisted of a total of 966 students, 500 of which were assigned to the VR condition (48% females, mean age = 13.9; ds = 1.3) and 466 to the NR condition (38% females; mean age = 13.5; ds = 1.3). Overall, 879 students filled out the questionnaires on T1 and 797 on T2 (87 students on T1 and 169 on T2 were absent from school on the days we administered the survey) (Fig. 1).

In summary, for the present study, we have four subgroups generated by the intersection of the following two conditions: the Experimental condition (Classrooms with voluntary peers and classrooms with nominated ones) AND the Peer educator condition vs the rest of the class. Specifically, in the VR condition, there are 101 peer educators (57% females, 50% middle school) and 399 classmates (45% females, 51% middle school). In the NR condition, there are 75 peer educators (36% females, 41% middle school) and 391 classmates (39% females, 34% middle school). In both groups of peer educators, the majority were Italian (84% among voluntary peer educators and 80% among the nominees).

In order to address our first goal, we used data from the first data collection (T1). Specifically, we compared the three subgroups: (1) voluntary peer educators, (2) nominated peer educators and (3) “All the classmates” who were the students from the two experimental groups not involved as peer educators. Excluding absentees on T1, we had 97 voluntary peer educators, 70 nominated peer educators and 712 classmates.

For our second goal, we used longitudinal data derived from both time points (T1 and T2). We compared changes over time in the two experimental groups (VR classes and NR classes) and in the peer educators vs the classmates condition.

### Measures

Bullying and victimization*.* The Florence Bullying-victimization Scales (FBVSs) (Palladino et al. 2016) were used. FBVSs consist of 20 items asking how often in the last couple of months the adolescents had experienced certain behaviours, either as perpetrators or victims (e.g. “I threatened someone”; “I was threatened”). A definition of bullying introduced the scale. Each item was evaluated along a 5-point scale from “never” to “several times a week.” The two subscales consist of 10 items each. In each set of data collection, the scales present acceptable internal consistency: for victimization at T1 Cronbach’s alpha is 0.77, and at t2 it is 0.79; for bullying at T1, it is 0.74, at T2 it is 0.77.

Defending Behaviour. We used an item of the Italian reduced version of the Participant Role Questionnaire (Menesini and Gini 2000; Salmivalli et al. 1996), a measure based on peer nominations. Students were asked to nominate an unlimited number of classmates as victim defenders (*Who are the boys or the girls who try to stop the bullying that a classmate is undergoing?*). For each student, we computed the total nominations obtained, divided by the number of students in the class, in order to assign a weighted score for each one. The estimates ranged from 0 to 1.

Popularity and Likeability. We used four items based on peer nominations. Students were asked to nominate an unlimited number of classmates as popular or unpopular (*Who are the most popular girls and boys in your classroom? Who are the least popular girls and boys in your classroom?)* and which ones they liked most and least (*Who are the boys and the girls that you like the most?—who do you enjoy the most or with whom do you spend your time? Who are the boys and the girls who you like the least—do you not enjoy or spend your time with?*). For each student, we computed the total nominations obtained, divided by the number of students in the class, in order to assign a weighted score for each one. The estimates ranged from 0 to 1. We used these continued variables in the following analyses.

### Overview of the Analyses

Analyses were conducted in SPSS. Given the non-normal distribution of victimization and bullying variables, we applied a logarithmic transformation and we used the transformed variables in all the subsequent analyses. *Attrition analyses* were carried out in order to evaluate whether adolescents with missing data at T2 differ significantly from adolescents with T1 and T2 data.

In order to test the comparability of the two experimental groups (baseline equivalence), we analysed the differences in the pre- test evaluations (Flay et al. 2005; Gottfredson et al. 2015). Specifically, we performed a set of multilevel mixed models on the target variables of our study (victimization, bullying, defending behaviour). The models used were 3-level random-intercept models (individuals within classrooms, within schools). A random-intercept model was fit to account for within-subject, within-classrooms, within-schools correlations. The fixed-effect portion of the model treated outcomes as a function of the experimental group condition (Classrooms with voluntary peer educators and classrooms with class-nominated peer educators). We used an alpha level of 0.05 for all statistical tests.

In order to address our first goal, a multivariate analysis of variance (MANOVA) was carried out, with the group condition (voluntary peers vs nominated peers vs classmates), gender and school grade (middle vs high school) as between-subject variables. Outcome measures were victimization, bullying, defending behaviour, popularity, unpopularity, likeability and unlikeability. When multivariate results were significant, univariate analyses were considered and post hoc comparisons were performed using a Bonferroni correction.

In order to address our second goal, we used linear mixed-effects models (MIXED) with full-information maximum likelihood (ML) estimation. MIXED procedure handles more complex situations, in which experimental units are nested in a hierarchy. Specifically, we (2a) tested whether the experimental condition (VR and NR) moderates the effectiveness of the NoTrap! program in reducing victimization and bullying, and in increasing defending behaviour; (2b) tested whether the longitudinal change in the target outcomes can be moderated by the role students played in the intervention (peer educators vs their classmates); (2c) tested for significant interactions between the experimental condition (VR and NR) and the role students played in the intervention (peer educators and their classmates); (2d), measured the effect sizes of the effects (pre-post change), comparing VR with NR method groups, and/or comparing peer educators and their classmates in each experimental condition when the interaction is significant.

We carried out three separate linear mixed-effect models, one for each outcome. The models used were 4-level (measurement occasion within individual, within classrooms, within schools) random-intercept models. A random-intercept model was fit to account for within-subject, within-classrooms, within-schools correlations. The fixed-effect portion of the model treated outcomes as a function of *time*, *experimental condition*, *peer educator’s role condition*, and the interactions between these variables. In order to obtain the most parsimonious model for each outcome, we included only significant interactions in the final model. As a second step, significant interactions were followed up by examining the outcome variables of each group across time. The random-effect portion of the model considers the random effects of subjects, classrooms, and schools.

Finally, for goal 2d, effect sizes (Cohen’s ds) of pre-post change were calculated. In particular, Cohen’s d was calculated as the adjusted group mean difference divided by unadjusted pooled within-group standard deviation. Specifically, we calculated the two experimental groups’ effect sizes, and, when significant, the effect sizes of the four subgroups generated from the interaction between the experimental condition and the peer educator’s role (voluntary peer educators vs their classmates vs nominated peer educators vs their classmates).

## Results

### Preliminary Analysis

The attrition analysis did not show any significant difference. In particular, the interaction between attrition by experimental group was not significant in relation to victimization (F_(1, 807.258)_ = .094; *p* = .760), bullying (F_(1, 816.853)_ = 3.178; *p* = .075) and defending behaviour (F_(1, 904.792)_ = .276; *p* = .600). Overall, it seems reasonable to assume that missing data across time are randomly distributed and not related to our outcome variables and can thus be ignored (Missing at Random- MAR). Consequently, for the present studies, we used all the information available across time. No significant differences were found for victimization (*F*_(1, 37.191)_ = .396; *p* = .245), bullying (*F*_(1, 49.153)_ = 3.936; *p* = .053) and defending behaviour *F*_(1, 37.001)_ = 1.518; *p* = .226) between the two experimental groups in the Pre-Test. This supports the assignment at *random*.

### First Goal

Descriptive statistics are reported in Table 1. Multivariate tests showed a significant effect of group (voluntary peer educators, class-nominated peer educators and classmates) for victimization, popularity, likeability and a trend for the effect on defending behaviour (Table 1). In particular, post hoc tests with the Bonferroni correction showed that voluntary peers had a significantly higher level of victimization than classmates. On the other hand, nominated peers had the highest level of popularity compared both to volunteer peers and all the classmates. They also had higher level of likeability and of defending behaviour than classmates.

**Table 1:** Descriptive statistics and tests between subject effects for STUDY I

	Volunteer peers (*n* = 95)		Nominated peers (*n* = 65)		Classmates (*n* = 650)		F, *p*, partial eta squared*		
	Mean	sd	Mean	sd	Mean	sd	Group	*Group by sex*	Group by school grade
Victimization	1.12	0.13	1.08	0.08	1.08	0.10	*F(2, 797) = 4.092; p = .017; η*^*2*^*= .013*	*F*(2, 797) = 1.331; *p* = .265	*F*(2, 797) = 0.831; *p* = .436
Bullying	1.06	0.08	1.1	0.12	1.07	0.09	*F*(2, 797) = 1.665; *p* = .190	*F*(2, 797) = 0.307; *p* = .736	*F*(2, 797) = 0.898; *p* = .408
Defending behaviour	0.09	0.09	0.10	0.09	0.07	0.09	*F*(2. 797) = 2.896; *p* = .*056*; *η*^*2*^ = .*007*	*F*(2, 797) = 0.686; *p* = .504	*F*(2, 797) = 0.966; *p* = .381
Popularity	0.13	0.15	0.20	0.20	0.11	0.16	*F*(*2, 797) = 9.221; p = .001; η*^*2*^*= .020*	*F*(2, 797) = 1.400; *p* = .247	*F*(2, 797) = 1.271; *p* = .281
Unpopularity	0.08	0.14	0.07	0.12	0.10	0.16	*F*(2, 797) = 2.442; *p* = .88	*F*(2, 797) = 0.593; *p* = .553	*F*(2, 797) = 1.518; *p* = .220
Likeability	0.20	0.11	0.23	0.12	0.18	0.12	*F(2, 797) = 5.511; p = .004; η*^*2*^*= .012*	*F*(2, 797) = 0.569; *p* = .566	*F*(2, 797) = 0.743; *p* = .476
Unlikeability	0.06	0.09	0.07	0.08	0.08	0.09	*F*(2, 797) = 1.269; *p* = .282	*F*(2, 797) = 0.238; *p* = .789	*F*(2, 797) = 2.403; *p* = .091

*In italics the significant effects (*p* < 0.05)

### Second Goal

Table 2 reported means and standard deviations of the target variables for the two waves of data collection for the two *experimental conditions*, and the *peer educator’s role conditions.*

**Table 2:** Descriptive statistics for the total sample and differentiated for experimental conditions and peer educator’s role condition

	Experimental condition 1: classrooms with volunteers peer educators				Experimental condition 2: Classrooms with peer educators nominated by classmates		
		Volunteer peer educators *N*, Mean(sd)	Volunteer peer educators’ classmates *N*, Mean(sd)	Total *N*, Mean(sd)	Nominated peer educators *N*, Mean(sd)	Nominated peer educators’ classmates *N*, Mean(sd)	Total *N*, Mean(sd)
Victimization	T1 (*N* = 814)	*N* = 88 1.117 (0.132)	*N* = 333 1.085 (0.102)	*N* = 421 1.091 (0.110)	*N* = 70 1.087 (0.077)	*N* = 323 1.0749 (0.090)	*N* = 393 1.077 (0.088)
	T2 (*N* = 745)	*N* = 80 1.090 (0.124)	*N* = 303 1.063 (0.098)	*N* = 383 1.068 (0.104)	*N* = 67 1.090 (0.119)	*N* = 295 1.0741 (0.117)	*N* = 362 1.077 (0.117)
Bullying	T1 (*N* = 817)	*N* = 88 1.058 (0.077)	*N* = 332 1.067 (0.078)	*N* = 420 1.065 (0.078)	*N* = 71 1.098 (0.118)	*N* = 326 1.0785 (0.097)	*N* = 397 1.082 (0.101)
	T2 (*N* = 743)	*N* = 80 1.036 (0.058)	*N* = 303 1.049 (0.064)	*N* = 383 1.046 (0.063)	*N* = 66 1.081 (0.114)	*N* = 294 1.0745 (0.091)	*N* = 360 1.076 (0.096)
Defending Behaviour	T1 (*N* = 941)	*N* = 92 0.088 (0.087)	*N* = 392 0.059 (0.076)	*N* = 484 0.064 (0.079)	*N* = 79 0.106 (0.099)	*N* = 378 0.0884 (0.103)	*N* = 457 0.0914 (0.102)
	T2 (*N* = 944)	*N* = 92 0.140 (0.119)	*N* = 392 0.096 (0.101)	*N* = 484 0.104 (0.106)	*N* = 79 0.140 (0.135)	*N* = 381 0.0956 (0.107)	*N* = 460 0.103 (0.114)

A significant interaction *time by* “*experimental condition*” has been found for victimization and bullying (Table 3). We also found a significant three-level interaction*—time by* “*experimental condition*” by *peer educator’s role condition—*for defending behaviour. This means that, with regard to victimization and bullying, in the two experimental conditions, the whole class (both peer educators and all the classmates) follows the same trend. Specifically, victimization and bullying significantly decrease over time only in classrooms with voluntary peer educators (victimization: *B* = 0.025; SE = 0.005; *p* < 001; bullying: *B* = 0.017; SE = 0.004; *p* < .001), while there were no changes over time in classrooms with nominated peer educators (victimization: *B* = − 0.000; SE = 0.006; *p* = .958; bullying: *B* = 0.005; SE = 0.005; *p* = .250).

**Table 3:** Mixed model predicting behavioural outcomes of victimization, bullying and defending behaviour

	Victimization			Bullying			Defending behaviour		
	*df*	*B* (SE)	*p*	*df*	*B* (SE)	*p*	*df*	*B* (SE)	*p*
Intercept	87.464	1.080 (0.010)	< .001	185.784	1.048 (0.008)	< .001	14.485	0.135 (0.017)	< .001
Time	766.441	0.024 (0.005)	< .001	731.078	0.017 (0.004)	< .001	942.569	− 0.052 (0.010)	< .001
Experimental condition	54.189	0.015 (0.010)	.151	60.655	0.029 (0.008)	.001	39.061	0.005 (0.023)	.507
Peer educator’s role condition	838.184	− 0.022 (0.008)	.006	828.054	0.000 (0.007)	.978	902.751	− 0.037 (0.009)	< .001
Time by experimental condition	767.472	− 0.025 (0.008)	.001	731.751	− 0.011 (0.006)	.048			
Time by experimental condition by peer educator’s role condition							1105.673	0.027 (0.006)	<.001
Residual variance		0.005 (0.000)	< .001		0.003 (0.000)	< .001		0.005 (0.000)	< .001
Subject: random intercept		0.005 (0.000)	.046		0.004 (0.000)	.023		0.001 (0.000)	< .001
Classroom: random intercept		0.000 (0.000)	< .001		0.000 (0.000)	< .001		0.004 (0.001)	< .001
School: random intercept		0.000 (0.000)	.249		^a^	.		0.000 (0.001)	.892

The final model both for victimization and bullying included the significant interaction *time by experimental condition*. For defending behaviour, the three-way interaction *time by experimental condition by educator’s role condition* was significant and included in the final model

^a^This parameter is set to zero because it is redundant

In relation to defending behaviour, it significantly increases over time in VR condition both for voluntary peers (*B* = − 0.055; SE = 0.010; *p* < .001) and their classmates (*B* = − 0.037; SE = 0.004; *p* < .001), whereas in NR condition, it increases only for nominated peer educators (*B* = − 0.030; SE = 0.012; *p* = .016), but not for their classmates (*B* = − 0.008; SE = 0.132; *p* = .132).

Comparing the two experimental conditions, effect sizes calculated using Cohen’s d showed stronger pre-post changes in classrooms with volunteer peer educators than for all the outcomes, compared to nominated recruitment conditions (respectively, a decrease of *d* = 0.14 vs no change (*d* = 0.00) for victimization; a decrease of *d* = 0.21 vs no change (*d* = 0.04) for bullying.

Regarding defending behaviour, following the significant interaction found in the previous analyses (experimental condition*time*peer educator condition), we compared the effect sizes of four subgroups. They showed that volunteer peer educators reported the strongest increase (*d* = 0.37); nominated peer educators and classmates of volunteer peer educators reported very similar effect sizes (respectively *d* = 0.16 and *d* = 0.14). Finally, for the classmates of nominated peer educators, there was no increase (*d* = 0.03).

## Discussion

The study contributes to the scientific debate on the effectiveness of peer-led models applied to an anti-bullying program (Gaffney et al. 2019b; Lee et al. 2015; Smith et al. 2012; Ttofi and Farrington 2011). The lack of consensus is due to the controversial findings of studies about programs that use a peer-led component (Palladino et al. 2016; Lee et al. 2015; Ttofi and Farrington 2011; Wong et al. 2011; Fonagy et al. 2009; Houlston and Smith 2009). A deeper examination of the different components involved in a peer-led approach could help us to understand *what works*, *for whom and under which circumstances* (Smith et al. 2012; Gottfredson et al. 2015). The present study aimed to address this call. In particular, the focus was on the peer educators’ recruitment strategy and on whether this factor may impact the program’s effectiveness. Specifically, we systematically compared two conditions: peer nomination recruitment and voluntary recruitment.

As a first aim, we found that the recruitment strategy led to having peer educators with different characteristics. In line with a previous study (Campbell and Marino 2009), when peer educators are nominated by classmates, the most popular and likeable students are chosen. It is interesting to notice that classmates seem to be able to identify the positive leaders in their class. In fact, nominated peer educators in our study as well are the highest in defending behaviour. This result enriches the literature about peer-led anti-bullying programs, as it gives us a description of peer educators chosen by peers. With regard to voluntary recruitment strategy, these results replicate what was found in a previous study with a different sample (Zambuto et al. 2019a). Voluntary peer educators are higher in victimization. This means that the decision to take on this role seems to be driven by direct involvement in bullying.

Looking at our second aim, results provide evidence that the strategy chosen to recruit peer educators may moderate the effectiveness of the intervention. We found that, despite 100% of classes in both conditions attended all the phases of the program, NoTrap! has been effective in reducing victimization and bullying and in increasing defending behaviour only in classrooms with the voluntary recruitment condition, both for peer educators and their classmates. On the contrary, in classrooms with the peer nomination recruitment condition, bullying and victimization remained stable, and defending behaviour increases only for peer educators, but not for their classmates.

The comparison between the two peer educators’ recruitment conditions gives us useful insights on the importance of an understudied aspect of peer-led models: the recruitment strategy. This single feature of a program can influence *under which circumstances* a peer-led model works or not. We can conclude that just *working with peers* may not be enough to make an intervention effective. As argued by Smith et al. (2012), different models and specific implementation procedures and features can make the difference between an effective peer-led program and an ineffective one. In line with this statement, our results suggest that, regardless of the strategy chosen, the recruitment phase should be well thought out, planned and tested in relation to other strategies, because it may have an impact on the program’s effectiveness.

Finally, regarding *for whom* the intervention is effective, we found that the voluntary recruitment condition was effective for the whole class. On the contrary, the nominated peer educators are not able to be agents of change for their classmates. In fact, while they increased the defending behaviour score, they seem unable to transfer this change to their classmates. This result is very relevant because in order to be considered effective, a peer-led model should pursue the objective of making peer educators able to act as agents of change in their class or group of reference.

Previous trials on the effectiveness of NoTrap! (Palladino et al. 2016) evaluated the classic version of the program, in which peer educators were volunteers. This means that the present study may be considered as a third independent quasi-experimental trial to confirm the effectiveness of the NoTrap! program in reducing bullying and victimization. Furthermore, while in the previous trials the authors considered only the global effects without differences between peer educators and their classmates, in the present study, the two target populations were tested separately. Results showed significant effects on both of them.

Moreover, the present study investigated a new outcome that had never been evaluated before. In fact, we found that NoTrap! increases the level of defending behaviour as well. The differences between VR and NR groups can explain why in the “peer nomination condition”, where there was no increase of defending behaviour in the classmates, we did not find any effect of the program also for bullying and victimization reduction.

In conclusion, the findings of the present study provide an important contribution to the debate on the effectiveness of *working with peers* in an anti-bullying program. The recruitment phase is a crucial step. Adopting voluntary recruitment versus peer nomination could lead to completely different levels of effectiveness of the program. This does not mean that voluntary recruitment should be preferred in all peer-led programs. On the contrary, our results suggest that the process of peer selection and recruitment must be kept into consideration in developing and validating a peer-led intervention.

### Limitation and Future Studies

Although the results are encouraging, we know that the absence of a control group is an important methodological limitation. In order to overcome this limitation, we randomly assigned participants to two experimental conditions, although we are aware that a control group would have strengthened our results. Additionally, schools and classes were selected using a self-selection inclusion process and we cannot exclude a potential selection bias. Moreover, a limitation of the study is the absence of a third recruitment strategy, “recruitment by adults.” The decision of not including this third condition was driven by the difficulties of using an adult-based approach in a model aimed to strengthen students participation. Further research should consider the characteristics and effectiveness of peer educators chosen by adults compared to voluntary recruitment. Finally, the sample size, the number of conditions and the complexity of the analyses cannot allow us to include gender in the model and clarify possible interactions with the conditions.

Besides, many questions remain open. For instance, future research could compare victims who decide to become peer educators to the ones that do not take on this role, in order to investigate whether students who decided to become peer educators are a “particular kind of victim,” different from the traditional ones. We know that they are victims who also perceived a high level of social support (Zambuto et al. 2019a), but it could be interesting to investigate whether they differ in other features, such as self-efficacy or resilience. Another interesting open research question is related to the motivation that supports peer educators in undertaking their role. Do they intend to help others or to reach a higher social status within their class?

Besides, further research could reveal which mediation mechanisms make voluntary peer educators more effective than nominated ones. We can speculate that the first ones have higher motivation for their task. A voluntary decision is intrinsically linked with higher motivation. Voluntary peer educators have been victimized in the past: this characteristic could make them more engaged with the program’s aims and tasks. Thanks to their direct involvement in bullying, they could also be higher in empathy toward other victims, and more motivated to learn useful coping strategies to escape from victimization to then transfer to their classmates as well. These two mechanisms remain the basis of NoTrap!‘s activities.

Another possible mediation variable may reside in the way in which voluntary peer educators are perceived by their classmates. A student who has been a victim could be seen as a reliable source of information in an anti-bullying program. Interviews and focus groups could be conducted with classmates in order to understand how they perceive students who voluntarily become peer educators vs the ones that are nominated for this role. On the contrary, nominated peer educators could be perceived as lacking motivation for this role and for their task. This could negatively impact their classmates’ engagement in the program workshops.

In relation to our data, future studies could also investigate whether the effects of the program on bullying and victimization could be mediated or moderated by the change of defending behaviour in the whole class.

Finally, it would be interesting to test if our results about the recruitment strategy can be extended to other areas of health interventions that adopt a peer-led model.

## Electronic supplementary material

ESM 1 (DOC 220 kb)
